# Arrest of the Development and Decalcification of the Enamel of Dentine

**Published:** 1893-03

**Authors:** Eugune S. Talbot

**Affiliations:** Chicago


### Arrests of Development and Decalcification of the Enamel and Dentine.

BY EUGUNE S. TALBOT, M.D., OF CHICAGO.

Read in the Section of Oral and Dental Surgery, at the Forty-third Annual Meeting of the American Medical Association, held at Detroit, Mich., June, 1892.

I wish to call your attention to a few observations which I have made in regard to a want of calcification and decalcification of the enamel and dentine. The conclusions arrived at are the result of the study of three cases which have presented themselves to me for treatment.

The first case is that of Miss C., 24 years of age. Her jaws have gradually come closer together, owing to a wearing away of the teeth. The front teeth have exceeded the back ones so that considerable space was noticed between her front teeth. The articulation was so poor that she was unable to chew her food and mastication was imperfectly performed with the vault of her mouth and tongue. Owing to the constant use of her tongue for purpose of mastication, hypertrophy had taken place to such an extent that her speech had become impaired. She was a constant sufferer from indigestion due to improper mastication, which was readily detected by the thick white coating on her tongue. Upon examination of her teeth, I found them of a dark yellowish color, containing grooves and pits, indicating arrest of development due to some of the eruptive fevers early in life. The bicuspids were the only teeth that had any semblance of normal contour, although the second molars were fairly well developed. None, however, were smooth like ordinary teeth. The roots of the second molars upon the right side of the jaw still remained, while the first molars had been extracted. The anterior teeth, both upper and lower, were worn away from one-third to one-half the length of their crowns. I judge that this was not due to friction by mechanical attrition for two reasons: first, the present teeth were very rough upon their cutting surfaces. If it had been due to attrition they would have been smooth; second, the roots of the second molars were smooth and polished. They did not occlude with the upper molars. Hence abrasion by attrition is wholly out of the question. In order to improve the appearance of the face and make the teeth serviceable for mastication, it was necessary to crown all the teeth except the six lower anterior ones. Upon scratching and shaving the crowns and roots, the enamel and dentine could be shaved off like horn or old cheese. The inclination of the crowns and roots of the upper anterior teeth when the jaws were in their proper position was such that the anterior and labial surface had to be shaved and ground down to such an extent that the pulp chamber was ground nearly to the cervical margin, without exposing the pulp, thus showing that the pulps had receded entirely from the crowns. This was also the case with the molars; no canals could be found in the roots. A sister of the patient had also similar teeth; she died at the age of five, of membranous croup. Her mother said that she had tried to have her children eat oatmeal for the purpose of improving the tooth structure, thus showing that she was not unmindful of the bad condition of the children's teeth. The father's teeth were in a similar condition although not quite so bad. The grandfather and grandmother on her father's side were both of a nervous temperament. Tartar accumulated early upon the teeth of all. The teeth of these girls erupted at an earlier age than usual.

CASE II, is that of a girl, aged 11 years, who was sent to me by her physician for an opinion as to the cause of her trouble and also for the purpose of treating her mouth and teeth. I found her an exceedingly nervous child, she would cry upon the slightest provocation. Her bones were small in diameter and she was rather short for her age. Upon examining her mouth I found the teeth covered with tartar of a bright yellow color. The tartar formed a continuous band extending from the second molar of one side to the second molar of the opposite side—this was upon both jaws. To such an extent had this accumulation taken place that it was impossible to locate the individual teeth until the tartar was removed. This was very difficult to accomplish because the slightest grating of the instrument upon the teeth would produce such a sensation upon her nervous system that she would nearly go into spasms. I could operate only about five minutes at a time. Upon the removal of the tartar, I found that the crowns, including the enamel and dentine were as soft and cut like horn. The tartar accumulates upon her teeth to such an extent that in two weeks' time, they will be completely covered. The child has occasionally nervous spells at which times all her teeth will ache. She suffered the most excruciating pain for about ten minutes after the impressions were taken for the models. No cavities were found in her teeth. The child takes plenty of nourishment but the phosphates and carbonates are not assimilated, which is also indicated by the small bones and nervous condition. Believing from the large amount of tartar which is excreted by the salivary glands that an excessive amount must also be excreted by the kidneys, I submitted a quantity of urine to Prof. Walter Haines, of Rush Medical College, for an examination. The following is his report: "From 100 cc. I obtain 0.0775 grams of the calcium phosphate ($Ca_3$ 2 $Po_4$), from which I calculate that the entire amount of calcium phosphate eliminated by the urine in 24 hours is 0.9168 grams. All the calcium in the urine existed as phosphate and none as carbonate. Guatier gives the average amonnt of calcium phosphate in the urine of an adult for 24 hours as 0.40 grams."

When we take into consideration, the age of this patient, and the fact that children are not in the habit of excreting the phosphates as grown people are, it will be found that this child is excreting through the kidneys four times as much as normal individuals at 30 years of age. None of the carbonates were present which shows that a large drainage was taking place from the system.

Case 3, is that of a gentleman, a lawyer, 42 years of age. He complained of sensitiveness of his teeth whenever food was taken into his mouth, he suffered no pain at other times. I found that all the teeth were arrested in their development, with pits and grooves running across the anterior teeth and clear around the bicuspids and first molars, and above and below these grooves, constrictions and irregularities of structure occurred, extending to and including the grinding surfaces. These conditions were the result of arrest of development due to the exanthemata—he informed me that he had had all the children's diseases. In all these teeth the enamel was imperfect and the cavities had been constantly excavated and filled. Upon examining these sensitive places in the molars, which were deep, I found that the structure cut like horn or old cheese-rind, and extended into the dentine an eight of an inch below the soft decay. The color was dark brown. We frequently find these spots of a darker hue in the molars, but still soft when cut with the excavator. No enamel or rather hard organized enamel is present. Believing from the depth of the cavity and the character of the pain that the pulp was exposed, I cut into the floor of the cavity with an excavator and finally with the bur until I was afraid of going through into the alveolar process without finding the canals. The tooth was afterwards filled with cement and has remained quiet for four months. The secretion of the mouth is alkaline.

CASE 4, is one which comes under the care of Dr. W. C. Barrett, of Buffalo, N. Y., and was presented before the Illinois State Dental Society in 1882. The following letter accompained the models. "The casts were made from impressions of the dentition of a young man, a resident of this city, of otherwise full development, in excellent general health, with no marks of inherited disease, and who comes of a sturdy race, the family occupying high social and business positions. Nearly two years elapsed between the periods of taking the lower and upper impressions, which accounts for an inexact occlusion.

When I first saw him, the possessor of this denture was about 18 years of age. The maxillæ, as might be seen in the casts, were well developed, and there was nothing approaching deformity, as the mouth presented a full and natural appearance, although, of course, no teeth were visible. I counseled him to refrain from any meddling, as his condition could not, in my opinion, be bettered by the dentists. At that age young men are very solicitous about their appearance, and annoyed I suppose at his peculiarity he applied to another practitioner who gave a different opinion, removed all the upper roots, and inserted a rubber denture which the young man is now wearing. I subsequently extracted some of the father's teeth and have made a microscopical examination of them.

It will be seen that there are absolutely no crowns to the teeth, their summits being for the most part upon a level with the gum and this was also the case with the deciduous dentition. The only vestige of enamel was some minute nodules or kind of bead-work around the cervical edges of two or three of the molars. The gums had a dense, glistening appearance, hard, unyielding, with apparantly less than normal vascularity, and seemed admirably adapted to assist in mastication, reminding one of the maxillary appearance presented by the graminivora that have incisors upon only one jaw. Of course there was nothing like a pulp chamber, but the roots of the teeth have nerve canals, which, however, are obliterated at the cervix. The exposed dentine before extraction of the teeth, seemed very dense and hard, and when tried with an excavator was about as sensitive as ordinary enamel. The color of the exposed ends of the teeth was of a rich, dark amber hue, approaching mahogany, and they were smooth and polished by attrition.

This peculiarity is not the result of disease, nor is there anything simulating the appearance of the atrophied crown which is sometimes the mark of arrested development, due to some functional derangement; it seems the result of hereditary transmission. The father had precisely the same dentition, though the son is the only one among several children, who has inherited this peculiarity. The father was also the only one in a large family who possessed this dentition. Beyond this I have no certain data, but I am informed that the mark has been persistent in the family as far back as they have any knowledge, although it has seldom appeared in more than one member at a time, some generations escaping entirely, while it cropped out occasionally in a collateral branch without being transmitted to descendants at all. In the direct line of descent it has, as well as I can determine, usually appeared in at least one member of the family, and that without material modifications. The young man of whose jaws these casts are fac-similes, may then reasonably be expected to transmit his denture to one out of a number of possible children, while the others will escape. His brothers and sisters may perhaps have children among whom will be found this lack of development, but the peculiarity will probably end there in their cases, while it is indefinitely continued in the direct line of descent.

When the father first presented himself to me, he had but two or three of these incomplete teeth left. These were worn down to mere points, and he had for years done his mastication upon the unprotected gum. The alveolus was so much absorbed that artificial substitutes had become a necessity and under my directions he was supplied with an entire new set. The roots, or the remainder of them which I removed, were of a dark mahogany color, due to staining of the dentine, and the nerve canal was entirely obliterated. They had once doubtless been of nearly the normal size, as are those of the son, but had been worn down to the point by mastication, having been gradually raised in their sockets to meet the demands of nature.

I made microscopical sections of some of the son's teeth and mounted them for examination. At the cervix of the teeth—the surface presented for mastication—very few marks of the dentinal tubules remained, they seeming to have been obliterated by an infiltration or deposition of calcareous dentinal matter. As I neared the apex of the roots the sections presented a more perfect dentinal arrangement, and the tubules more of the usual appearance though the whole structure of the tooth was loose and showed numbers of minute chambers, some of them apparently communicating by what appeared to be abnormally large dentinal tubules. The nerve canal, entirely obliterated upon the grinding surface, (though from the direction and arrangement of the few tubules visible it had once existed) farther towards the apex of the root made its appearance and was of nearly normal size. The ends of the roots were well formed, and the apical foramen had the appearance of fully developed and well grown teeth. The cementum was much less in proportion than is usual, the dentine at most being invested with but a very thin layer, but what there was, presented no unusual appearance. The enamel, which was found in minute nodules at the cervix of two of the teeth, was extraordinary in appearance. It did not present the usual relation to the dentine, and at the junction of the two there was not the frequent reaching over of some of the tubules. The enamel rods had not the usual regular appearance, but the substance of the enamel seemed twisted and contorted. It existed in minute aggregations, and seemed to have little of the usual relations with dentine. The most of the teeth had no enamel whatever. I can do little by way of accounting for this abnormal dentition. The entire absence of enamel in most of the teeth must have been due to a lack of enamel organ, or to its functional inactivity. The teeth erupted about the usual period, though perhaps a little later than usual. They have never shown any signs of caries, those of the father wearing out without decay, and those of the son being perfectly sound and quite exempt from disease. The case has been one of interest to me, and I should be glad to know if any other dentist has met with a like peculiarity."

Such conditions are frequently observed in connection with one or two or even three and four teeth. I can recall a number of cases where the lower central and the lateral incisors of both sets are in the same condition, while all the other teeth are normal. I can also recall a case where the bicuspids upon both sides are in the same condition and the other teeth are normal; and also other cases where the first molars upon both sides are in the same condition and also the roots of the second lower right molar, although I am not quite certain of this; the other teeth are normal. I have frequently seen and you have all observed the first permanent molars in that soft condition, contracted at different points, presenting holes and grooves which run in all directions. The decay extends deeply and is of a dark brown color resembling spunk or the decay of the heart of a tree; the tooth is exceedingly sensitive to touch and painful when food of different qualities comes in contact with it. The secretions of the mouth are usually perverted, and when these teeth are excavated, the softness of the enamel and dentine is readily noticed. The pulp recedes frequently, obliterating the pulp chamber.

The roots of the case illustrated by Dr. Barrett, seem to be covered at the cervical margin with dentine, which would also indicate that the pulp had also receded. I have observed this in the roots of molars, bicuspids and incisors when in a similar condition.

When this condition takes place in old people the enamel and dentine become exceedingly hard, and no sensation is experienced when the tooth is excavated. You will therefore observe that while the recession of the pulp is precisely the same in both cases the results are directly opposite. We have all observed teeth of a dark color, and so worn down that occlusion is impossible, while the teeth anterior and posterior to them, are in a healthy condition—this is frequently the case with the first permanent molar.

Their surfaces are smooth and polished. The color of the crowns of these teeth are not unlike that of the roots and teeth already illustrated in the cases presented.

I should class under the head of abrasion and erosion cases where one, two or even all the teeth are affected and where the color of the enamel and dentine is normal. Many theories have been advanced of the etiology of abrasion and erosion but I think that all these cases can to a certain extent be classed under the same condition, namely, primarily a want of calcification and a decalcification of the enamel and dentine. We frequently find that the first permanent molar and sometimes the second and third are worn away, and all the other teeth are normal. These mouths sometimes present strange conditions. First, decay is rarely present. Dr. Barrett very aptlv says in his case, "that the teeth showed no signs of decay, those of the father wearing out without decay. The secretion of the mouth is most always alkaline and quantities of tartar are usually present. In ordinary mouths when the secretions are acid decay would take place because of the recession of the pulps—u-ually tartar in large quantities is present. These conditions render decay almost impossible. These teeth are always sott, cut readily under the bur and the pulps recede so that in many cases the pulp chamber is entirely filled. The anterior teeth upon the upper and lower jaw will wear away frequently leaving a space of from one-fourth to three fourths of an inch between them, while the teeth posterior to them occlude.

The enamel although nearly twice as dense as the dentine wears away as readily as the dentine. What conclusion can we draw from these facts? We have already shown that in some cases the phosphates and carbonates are not assimilated. This to my mind is the cause of erosion and abrasion. We instinctively make the remark to a patient that it is better for teeth to erupt late because the tooth structure will be of a better quality. I have frequently observed this in irregularities of the teeth—When these teeth are slow in coming through, they are larger, more dense in structure and set more deeply in the jaw. The reverse is the case when they erupt early. The structure of the tooth is not well nourished. This condition can also occur in utero, the mother not giving sufficient nourishment to her child. Dr. Marshall in his paper entitled "The Oral Cavity of Pregnant Women" read at Newport, 1889, alludes to "certain cases of pregnancy in which the nutrition was impaired and as a result the bones and dentine became abnormally soft, and that thus this softening of the dentine predisposes the teeth to decay." The child after birth may be poorly nourished and thus the bone, teeth as well as other tissues of the body suffer as a result. You are all familiar with the fact that the teeth suffer from a want of nourishment, and arrest of development will take place when the child is afflicted with the constitutional diseases. These teeth are soft and when the enamel is lost decay attacks them. After the teeth are formed they frequently decalcify. This is well illustrated in the case of pregnant women—the teeth seem to lose their vitality and decay early. If the teeth disintegrate under these conditions why will they not in constitutional diseases, as consumption, typhoid fever, etc? We know that this is the case so far as decay is concerned. I have called your attention to the fact that in some cases especially in children between 12 and 20 the phosphates and carbonates do not assimilate and hence there is a softening of the teeth and bones. We find that these substances which are taken in the system in the form of food for the nourishment of the body are deposited, unorganized upon the teeth, being excreted by the salivary glands. These substances are also excreted by the kidneys. The brain is not properly nourished, and as a result the child becomes very nervous, or again the child may possess a very active brain and the phosphates are entirely used up in its nourishment, the bones and teeth suffering in consequence. I have plenty of material that could be brought forward to sustain this theory, but the paper is now so long that I will not weary you with any further data. I have tried to find some mode of treatment but at the present time have been unable to lay down any fixed rule. Plenty of outdoor exercise and good nutritious food are about all the measures that can be suggested. It is possible that if the child lives long enough he may outgrow this want of assimilation.

DISCUSSION.

Dr. W. C. Barrett: We should try to determine the reason of the want of enamel in these cases. In one case there was a total lack of crowns to the teeth, and no enamel at all, except some little nodules at the gingival portion. In my report, quoted by Dr. Talbot, I stated that the dentinal tubules were not entirely normal. I believe this was because there were no crowns to protect them, and being normal at first, the modifications were caused by the want of the protection of enamel. The roots were perfect and mastication was performed upon the tops of the roots. Of course there was a call for this absence of enamel. Could it be an entire failure of the enamel-membrane derived from the epiblast while the mesoblast performed its office correctly? The deciduous teeth of these patients had some kind of crowns. I do not know and could not learn just how near to normal they were.

We should take up and make a study of the pits and cracks often found in enamel. No one has as yet demonstrated the reason for them. They have been ascribed to the diseases of childhood, but if that is true why do they not occur all over the enamel? The enamel is all formed at one time, and these pits and marks should be in all parts of it. We all know that this is not generally the case, and we cannot prove that they are connected with the eruptive diseases of childhood. If every child who had eruptive diseases had these defects in its teeth, then it might prove that they were the cause. But it is not so.

Dr. Gish reported a case of a child, seven years old whose temporary teeth were in a condition very much resembling the teeth described by Drs. Talbot and Barrett, only that they had had crowns. When he first saw the case, all of the deciduous teeth were worn off even with the gums, and the roots were of a polished mahogany-brown appearance. He watched the case with a great deal of interest. He had to remove the roots of the premolars, as they were so firm that the permanent teeth could not erupt. When the permanent teeth did come in, they were quite perfect. What factor could cause such a condition in the deciduous teeth which still would not affect the permanent set?

Dr. Fletcher asked Dr. Barrett how many generations of the family—the subject of his report—he had positive knowledge of.

Dr. Barrett said he had positive knowledge only of the father and son. As for the preceding generations, he knew nothing only that it was an accepted tradition in the family that there had for a long time been one or more with short teeth in each generation.

Dr. Allport spoke of three girls in one family, who were patients of his, whose teeth were quite short and of a light slate color; in two of the girls the back teeth only were affected, in the third, even the front teeth were of the same color. He learned that the grandfather had teeth of the same character, though the boys in the same family had perfectly natural and well-formed teeth. The peculiarity was evidently hereditary.

Dr. Talbot said, in closing the discussion, he wished to call the attention of the section to the frequency of cases of a want of assimilation of the phosphates and carbonates in the food, and the consequent lack of these elements in the teeth, bone, and brain. We will find a child, say of twelve or fourteen years,—likely a girl,—very nervous; eats everything, beefsteak, milk, etc., yet is so nervous that she cannot control herself,—perhaps cannot hold her urine. She will be a bright child, with small bones. Upon examination her teeth will be found full of tartar, and her urine loaded with carbonates and phosphates. This material, so badly needed to supply the bones, teeth, and nervous system, is not assimilated, but is excreted by the salivary glands and in the urine.

Similar conditions are found in pregnancy; the teeth decay more rapidly than they would otherwise, and if a limb is broken the bones cannot be gotten to unite. These cases show that the phosphates and carbonates are appropriated by the fœtus. We know, too, that in many diseases the teeth do not receive sufficient nourishment. The teeth show erosions arising from the constitutional condition which produces softening, and the attrition of the food and the adjacent parts wear the teeth away. Dr. Truman says that in erosion the structure of the teeth is harder than usual, but I think it is softer.

We all have these patients, but none of us know how to treat them successfully. We are advised to take the child from school, keep her out of doors, on a farm, with horseback or bicycle riding. We will try all these and many other measures, but with little prospect of success. The probability is that such a child will die of consumption within a few years.

Another condition of the system which affects the teeth is gout. A gouty patient will almost invariably, if not quite, show a softening and erosion of the teeth.

Dr. Fletcher said he had a case, a patient about forty-five or fifty years old, whose incisors were entirely denuded of enamel on the labial side. The appearance was not as if it had dissolved away, but rather as if it had split off, as the edges were distinct.

Dr. Clifford asked if there was any peculiar diathesis in this case.

Dr. Fletcher said he did not know of any.

Dr. Barrett reported a case somewhat similar, with the enamel all gone from the labial surface, leaving it smooth. The teeth came to a sharp edge and crumbled away. He did not know what to do with them unless he put crowns on them. The enamel on the lingual surface was intact. They were just the reverse of the teeth of rodentia.

On motion, the subject was passed and Dr. J. L. Gish read a paper on "The Diseases of the Gums."

Dr. Gish's paper included a classification of the various pathological disturbances of nutrition as they are expressed in the soft tissues of the mouth. He described clearly and fully the several types of inflammation, from simple acute to chronic suppurative inflammation, as well as the cellular changes expressed as morbid growths. The pathological processes, as well as the clinical appearances in their different phases, were fully set forth, and the paper was a fair *resume* of present knowledge in this department of pathology.

Its great length, and our lack of available space, coupled with the fact that we have recently presented our readers with similar matter somewhat *in extenso*, precludes a fuller abstract.

Dr. Fletcher related an experiment, which he had made, to show the influence of the faradic current in the circulation. He isolated the mesentery of a frog, and under the microscope watched the circulation until stasis had set in in a few of the vessels; then by turning on the faradic current he cleared up the whole zone. The shock stirred up the white blood-corpuscles and started up the circulation, He had found the use of the current in diseases of the gum very satisfactory, as described in the paper.

Dr. Curtis said the essayist had ably described the pathological conditions of diseased gums, and he regretted that the treatment had not been described more fully. He believed electricity was a great remedy for these diseases, and as we come to understand the use of it better, we will be better able to treat them.

Dr. Marshall, said in regard to the use of the electrical current, if the positive pole is placed over a location where hyperæmia exists, and the negative pole over the base of the brain, the hyperæmia will be decreased. If the poles are reversed, the hyperæmia will be increased. His treatment had been mostly in cases of hyperæmia of the pulp. When the tooth does not positively pain the patient, but feels as if toothache were imminent, by applying the current and repeating for a day or two the condition will be passed, and the necessity for further treatment averted.

